# Promising application of monoclonal antibody against chikungunya virus E1-antigen across genotypes in immunochromatographic rapid diagnostic tests

**DOI:** 10.1186/s12985-020-01364-4

**Published:** 2020-07-02

**Authors:** Keita Suzuki, Ralph Huits, Juthamas Phadungsombat, Aekkachai Tuekprakhon, Emi E. Nakayama, Riemsdijk van den Berg, Barbara Barbé, Lieselotte Cnops, Rummana Rahim, Abu Hasan, Hisahiko Iwamoto, Pornsawan Leaungwutiwong, Marjan van Esbroeck, Mizanur Rahman, Tatsuo Shioda

**Affiliations:** 1 Research Institute for Microbial Diseases, Osaka University, Suita, Japan; 2 POCT Products Business Unit, TANAKA Kikinzoku Kogyo K.K, Hiratsuka, Japan; 3 Department of Clinical Sciences, Institute of Tropical Medicine, Antwerp, Belgium; 4 Mahidol-Osaka Center for Infectious Diseases, Mahidol University, Bangkok, Thailand; 5 Department of Microbiology and Immunology, Mahidol University, Bangkok, Thailand; 6 Landslaboratorium Aruba, Oranjestad, Aruba; 7 Apollo Hospitals Dhaka, Dhaka, Bangladesh

**Keywords:** Chikungunya virus, E1 protein, Rapid immunochromatographic RDT, Monoclonal antibody, ECSA, Asian genotype

## Abstract

**Background:**

Three different genotypes of chikungunya virus (CHIKV) have been classified: East/Central/South African (ECSA), West African (WA), and Asian. Previously, a rapid immunochromatographic (IC) test detecting CHIKV E1-antigen showed high sensitivity for certain ECSA-genotype viruses, but this test showed poor performance against the Asian-genotype virus that is spreading in the American continents. We found that the reactivity of one monoclonal antibody (MAb) used in the IC rapid diagnostic test (RDT) is affected by a single amino acid substitution in E1. Therefore, we developed new MAbs that exhibited specific recognition of all three genotypes of CHIKV.

**Methods:**

Using a combination of the newly generated MAbs, we developed a novel version of the IC RDT with improved sensitivity to Asian-genotype CHIKV. To evaluate the sensitivity, specificity, and cross-reactivity of the new version of the IC RDT, we first used CHIKV isolates and E1-pseudotyped lentiviral vectors. We then used clinical specimens obtained in Aruba in 2015 and in Bangladesh in 2017 for further evaluation of RDT sensitivity and specificity. Another alphavirus, sindbis virus (SINV), was used to test RDT cross-reactivity.

**Results:**

The new version of the RDT detected Asian-genotype CHIKV at titers as low as 10^4 plaque-forming units per mL, a concentration that was below the limit of detection of the old version. The new RDT had sensitivity to the ECSA genotype that was comparable with that of the old version, yielding 92% (92 out of 100) sensitivity (95% confidence interval 85.0–95.9) and 100% (100 out of 100) specificity against a panel of 100 CHIKV-positive and 100 CHIKV-negative patient sera obtained in the 2017 outbreak in Bangladesh.

**Conclusions:**

Our newly developed CHIKV antigen-detecting RDT demonstrated high levels of sensitivity and lacked cross-reactivity against SINV. These results suggested that our new version of the CHIKV E1-antigen RDT is promising for use in areas in which the Asian and ECSA genotypes of CHIKV circulate. Further validation with large numbers of CHIKV-positive and -negative clinical samples is warranted. (323 words).

## Introduction

Chikungunya virus (CHIKV) is a mosquito-transmitted alphavirus belonging to the family *Togaviridae*. This pathogen was first isolated in Tanzania, East Africa, in 1952 [1, 2]. Since then, CHIKV has caused sporadic outbreaks throughout the African and Asian continents. Although only one serotype CHIKV exists, the virus is classified into three genotypes named after the geographical location where the respective genotype was first recognized: East/Central/South African (ECSA), West African (WA), and Asian [3, 4]. CHIKV was considered a neglected tropical pathogen until a large outbreak was reported on Indian Ocean islands in 2005. The outbreak affected 244,000 individuals, one-third of the total population in this region [5, 6] . Sequence analysis revealed a specific amino acid change that rendered the virus infective to *Aedes albopictus*; this clade of the ECSA genotype therefore was designated as the Indian Ocean Lineage (ECSA-IOL) [6, 7]. Subsequently, ECSA-IOL also was identified in India [8] and South East Asia (including Thailand [9], Cambodia [10], and Malaysia [11]), and was detected for the first time in European countries (including Italy [12] and France [13]). In contrast, non-IOL ECSA-genotype CHIKV was detected in Brazil in 2013 [14, 15]. Asian-genotype CHIKV subsequently gave rise to a novel clade, designated as Asian/American [16, 17]. This clade has become a public health problem in the Caribbean islands and the Central American mainland [18, 19]. Worldwide, approximately one billion people are estimated to live in areas at risk of CHIKV outbreaks [20].

More than 75% of CHIKV-infected individuals develop acute febrile symptoms such as high fever, headache, and muscle and joint pains [21, 22] that are similar to those observed in patients infected by other mosquito-borne viruses such as dengue dengue and Zika viruses [23]. However, the CHIKV illness often is associated with prolonged and incapacitating arthritis; for this reason, large CHIKV epidemics have considerable economic consequences, highlighting their significant public health impact. To distinguish CHIKV infection in the acute (viremic) phase, several nucleic acid detection methods are considered the gold standards [24]. However, immunochromatographic (IC) rapid diagnostic tests (RDTs) are user-friendly and easy to store without the need to maintain a cold chain, while facilitating early diagnosis. Such RDTs are expected to increase the accessibility of laboratory diagnosis of CHIKV infection. Previously, Okabayashi et al. reported a prototype IC RDT with high sensitivity (68 out of 76, 89.4%) and specificity (34 out of 36, 94.4%) against clinical samples known to contain ECSA-genotype CHIKV in Thailand and Laos [25]. That RDT also showed high sensitivity (74 out of 79, 93.7%) and specificity (42 out of 44, 95.5%) in India [26]. However, another trial of that RDT with confirmed clinical samples of Asian-genotype CHIKV from the island of Aruba revealed low sensitivity (10 out of 30, 33.3%) [27].

We previously showed that CK47, one of the monoclonal antibodies (MAbs) used in the RDT described by Okabayashi et al., has a limited ability to recognize the Asian-genotype E1 protein [28]. Notably, the Asian-genotype CHIKV encodes an aspartic acid (D) at residue 284 of the predicted E1 protein (residue 350 of the 6 K-E1 polyprotein), a position that is encoded as a glutamic acid (E) in the predicted E1 proteins of certain ECSA-genotype CHIKVs [28]. This polymorphism critically affected the utility of that diagnostic tool across the various CHIKV genotypes [17]. To improve the sensitivity and specificity of the IC RDT, we obtained several anti-CHIKV MAbs targeting the E1 and capsid (CA) proteins of CHIKV [29]. These MAbs were shown to exhibit broad reactivity towards all genotypes of CHIKV, and some of these MAbs lacked cross-reactivity to other alphaviruses, including eastern, western, and Venezuela equine encephalitis viruses. Here, we report the development and evaluation of an improved CHIKV antigen-detection diagnostic test that incorporates these new MAbs.

## Materials and methods

### Screening of MAbs against CHIKV E1 and CA

To select anti-E1 MAbs suitable for use in an IC RDT, we screened 7 anti-E1 MAbs (3D11, 9E3, 11E11, 13H11, 15B2, 19B8, and RC-5) generated previously [29] by adsorbing each anti-E1 MAbs onto nitrocellulose membranes. Six of these MAbs (excepting 15B2) were conjugated with gold nanoparticles (AuNPs); 15B2 was excluded because that MAb is isotype IgG2b [29], which is difficult to conjugate with AuNPs (unpublished observations). All combinations of anti-E1 MAbs were tested by placing dipsticks in microcentrifuge tubes or the wells of a 96-well plate containing (per tube or well) 60 μL of extraction buffer and 30 μL of formaldehyde-inactivated culture supernatant of CHIKV S27-infected Vero cells. After 15 min, a C10066–10 IC Reader (Hamamatsu Photonics K.K., Japan) was used to quantitate the intensities of the signals, and results were expressed as signal/background (the intensities of the signals without CHIKV antigens) ratios. This procedure was repeated another three times using recombinant CHIKV E1 protein (Aalto Bio Reagents Ltd., Dublin, Ireland), culture supernatant from Vero cells infected with Sendai virus expressing Asian CHIKV CK12 6 K-E1protein [29], and culture supernatant of CHIKV CP10-infected Vero cells. In the case of anti-CA MAbs, all five MAbs (24B3, 26A2, 32A3, 37C7, and 41G5) [29] were able to be conjugated with AuNP and evaluated by the procedure described for anti-E1 MAbs using culture supernatant of CHIKV CP10-infected Vero cells as the antigen.

### Assembly and evaluation of IC RDT

The details of components of the 1st version (version A) have been described previously [25]. Briefly, the rapid IC RDT incorporated two mouse anti-CHIKV MAbs: CK47 was immobilized onto the membrane at the test line and used for CHIKV antigen-capture; CK119 was conjugated to AuNPs and placed at the conjugated pad by TANAKA Kikinzoku Kogyo K. K, Japan. The 2nd (versions B, C, D, E, F, M, N) and 3rd (version O) -generation IC RDTs were assembled with the combinations of MAbs shown in Table 1.

**Table 1:** List of antibody combinations

Version	Detection antibody (gold labeled)	Capture antibody (membrane bound)	Target
A	CK119	CK47	E1
B	13H11	3D11 + 15B2	E1
C	41G5	26A2	Capsid
D	41G5	24B3	Capsid
E	13H11 + 11F11	15B2	E1
F	26A2 + 41G5	24B3	Capsid
M	26A2 + 41G5	32A3	Capsid
N	13H11 + 26A2 + 41G5	15B2 + 32A3	E1 + capsid
O	13H11 + CK119	3D11 + 15B2 + CK47	E1

Thirty microliters of serially diluted culture supernatant containing CHIKV or CHIKV-pseudotyped lentiviral vector at various concentrations were mixed with 60 μL IC RDT extraction buffer in a tube. The IC dipstick then was inserted into the tube of diluted supernatant to start the reaction. After 15 min, the appearance of the control and test lines was assessed. An IC Reader also was used to quantitate the intensities of the test lines; values were expressed as milli-absorbance units (mAbs).

### Cells and viruses

African green monkey kidney epithelium (Vero) cells (ATCC CCL-81) and baby hamster kidney (BHK) cells (ATCC CCL-10) were maintained in Minimum Essential Medium (MEM; Life Technologies, Inc.) supplemented with 10% (v/v) heat-inactivated fetal bovine serum (FBS; Life Technologies, Inc.). Human Embryonic Kidney (HEK293T) cells were maintained in Dulbecco’s Modified Eagle Medium (DMEM; Life Technologies, Inc.) supplemented with 10% (v/v) heat-inactivated FBS. These cells were maintained at 37 °C in a 5% CO_2_ environment. For culturing cells that had been infected with CHIKV, the concentration of FBS in the medium was reduced to 2%. *Aedes albopictus*-derived C6/36 cells were maintained at 28 °C in L-15 medium (Life Technologies, Inc.) supplemented with 2% heat-inactivated FBS and 0.3% tryptose phosphate broth.

The ECSA-genotype CHIKV strain CP10 was isolated during a 2010 outbreak in Thailand [30, 31]; Asian-genotype strains ARUBA-15801125 (NCBI Accession Number LC500216), ARUBA-15800567 (LC500220), and ARUBA-15801654 (LC500221) were isolated from the sera of patients in Aruba [27, 32, 33]. Sindbis virus (SINV; strain R68), another alphavirus, was propagated in BHK cells. CHIKV strains were propagated using Vero and C6/36 cells. For viral titration, a standard plaque assay was performed [34].

### CHIKV-pseudotyped lentiviral vector

The production of the CHIKV-pseudotyped lentiviral vectors was performed as described previously [35], with some modifications. Briefly, the process employed three essential plasmids: a pCAGGS MSII derivative that harbors a DNA cassette encoding the Asian-genotype CHIKV E3-E2-6K-E1 polyprotein corresponding to the CK12–686 strain [28]; pLenti CMV Puro LUC (w168–1), which carries a reporter gene encoding firefly luciferase (Addgene, Cambridge, MA); and psPAX2, a lentivirus packaging vector. All three plasmids were transfected into HEK293T cells using polyethelenimine (Polysciences, Inc., Warrington, PA) [35]. Culture medium was replaced with fresh medium after 6 h; the spent medium then was collected at 48 h after transfection. The titer of lentivector was determined using the RETROtek HIV-1 p24 Antigen-ELISA kit (Zeptometrix; Buffalo, NY), according to manufacturer’s instructions. For production of WA- and ECSA-genotype E1s, we used pCAGGS MSII derivatives encoding (respectively) the E3-E2-6K-E1 sequence of WA-genotype strain 37,997 or a chimeric E3-E2-6K-E1 sequence composed of sequences from strain 379,971 E3-E2 and strain CP10 6 K-E1.

### Clinical samples

The Asian-genotype CHIKV serum panel was obtained for evaluation of the 2nd-generation version-B RDT from CHIKV-specific RT-PCR-positive (*n* = 26) and -negative (*n* = 54) samples of patients who had presented with febrile illness during the 2014–15 CHIKV epidemic in Aruba. These sera had been collected at 3–10 days (median 7 days) after the onset of symptoms. Median Ct value (and its interquartile range, IQR) of RT-PCR-positive samples were 35.29 (34.22–35.50). Forty samples were shown to be anti-CHIKV IgM- or IgG-positive by testing in Aruba using the Anti-Chikungunya Virus IgM/IgG IIFT assay (Euroimmun, Lübeck, Germany). Among this sample subset, 34 were anti-CHIKV IgM-positive and 31 were IgG-positive (25 were both anti-CHIKV IgM- and IgG-positive). Samples then were stored at − 80 °C until shipped on dry ice to Institute of Tropical Medicine, Antwerp (ITM), where CHIKV-specific RT-PCR testing was performed [32]. After this testing, these specimens were stored at − 80 °C until evaluation by the E1-antigen IC RDT (i.e., after being subjected to a total of 2 freeze-thaw cycles). The protocol was approved by the Institutional Review Board of the ITM (1013/15).

A second set of clinical samples was obtained from the Apollo Hospitals Dhaka, Bangladesh. In 2017, a sudden increase in febrile cases was observed in Dhaka City, Bangladesh. Between July 2017 and February 2018, 1688 patients visited Apollo Hospitals Dhaka, with acute onset of fever (within 7 days from onset), myalgia, arthralgia, and headache; some patients also experienced a maculopapular rash and/or gastrointestinal symptoms. Out of 1688 febrile cases, 643 (38.1%) and 269 (15.9%) were laboratory-confirmed as chikungunya fever and dengue fever, respectively, by RT-PCR with the simultaneous detection and differentiation of CHIKV and dengue virus (DENV) (Fast-Track Diagnostics, Luxembourg). Notably, the sequences of CHIKV in this season in Dhaka (NCBI Accession No. LC364266-LC364269) were reported as ECSA genotype [36], encoding a 6 K-E1 polyprotein with an E at residue 350 (corresponding to residue 284 in the mature E1 protein). These CHIKVs lacked an alanine-to-valine substitution at residue 226 of the E1. CHIKV PCR-positive or DENV PCR-positive sera were stored at − 80 °C. The titer of serum IgM against CHIKV was measured using an ELISA kit (MBS495200) purchased from MyBioSource (San Diego, CA, USA). One hundred CHIKV RT-PCR-positive and 100 DENV RT-PCR-positive sera were used for evaluation of the 3rd-generation version-O RDT. The median Ct value (IQR) of CHIKV RT-PCR-positive samples was 17.02 (14.89–19.92). The study proposal was approved by the research and ethics committee of Apollo Hospitals Dhaka (ERC 16/2018–1).

## Results

### Selection of MAbs against CHIKV E1 and CA in the 2nd generation of RDT

We began our study by performing screening of the 7 anti-E1 MAbs that we had generated previously [29]. We used four different antigens for this screen, as described in Materials and Methods. Mean and standard deviations of signal/background ratios are shown in panel A of Additional file 1. Thirty-three of the 36 tested combinations yielded low signal/background ratios in the context of immunochromatography. One combination showed high levels of variation and only two combinations showed signal/background ratios above 2.0. In the case of 5 anti-CA MAbs [29], more than half of the combinations (16 out of 20) gave signal/background ratios above 2.0 when tested against the culture supernatant of CHIKV CP10-infected Vero cells (panel B of Additional file 1). Therefore, we chose the combinations of anti-E1 MAbs (IC RDT versions B and E) and anti-CA MAbs (IC RDT versions C, D, F, and M) to construct trial IC RDTs (Table 1).

### Sensitivity of the 2nd-generation IC RDTs to ECSA- and Asian-genotype CHIKV isolates and CHIKV envelope-pseudotyped viruses

**Fig. 1:**
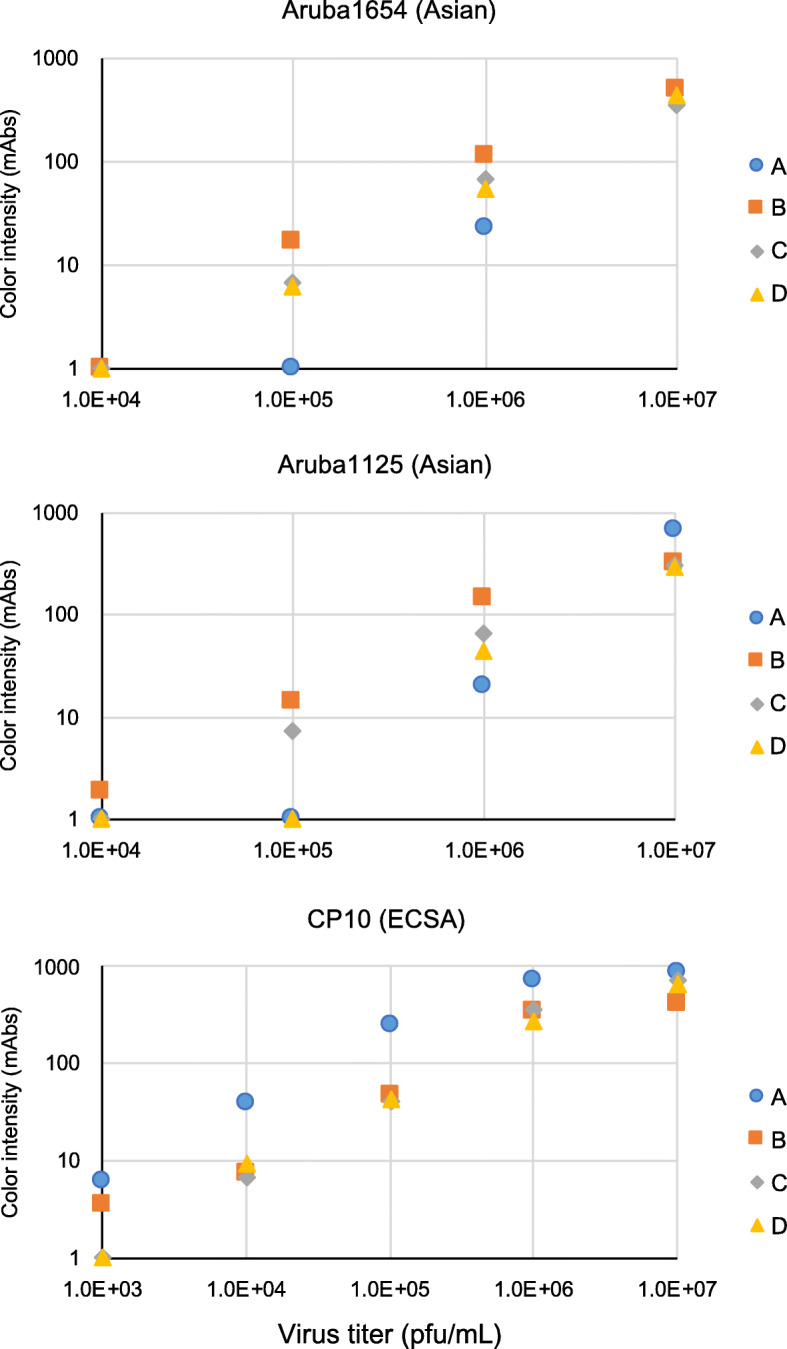
RDT reactions to Asian-genotype CHIKV strains ARUBA-15801654 and ARUBA-15801125, and to ECSA-genotype strain CP10. ARUBA-15801654 (ARUBA1654) and ARUBA-15801125 (ARUBA1125) were propagated in C6/36 and CP10 was propagated in Vero cells. The x-axis denotes viral titer in plaque forming units (PFU) /mL. Blue diamonds, orange squares, gray triangles, and Xs indicate version-A, −B, −C, and -D RDT measurements, respectively. The y-axis indicates the intensity of the test line (milli-absorbance units; mAbs)

To evaluate the reactivity of these new CHIKV antigen detection RDTs, we used four dilutions of ECSA- and Asian-genotype viruses, at titers of 10^7, 10^6, 10^5, and 10^4 PFU/mL. The limit of visible detection (i.e., with naked eyes) of CHIKV protein was 15 m-absorbance units (mAbs) of color intensity as measured by an IC Reader. All rapid IC strips gave valid results, as indicated by the presence of the control lines. Quantitative measurement of the intensities of the test lines revealed that the E1 antigen-detecting RDT version B showed the best performance in detection of Asian-genotype CHIKV (Fig. 1, Additional file 2). At higher concentrations of CHIKV, the gold-conjugated antibody in versions B and E tended to aggregate, thereby impeding quantifiability (Fig. 1 and Additional file 2). In the case of ECSA-genotype CHIKV, the previous version-A RDT exhibited the best performance (Fig. 1). In contrast to the E1 detection RDTs, CA detection RDTs displayed similar reactivity to both CHIKV genotypes. Nevertheless, none of the CA detection RDTs was as sensitive as the E1 detection RDTs, especially at 10^5 pfu/mL of CHIKV. Furthermore, addition of anti-CA antibodies to an E1 detection RDT failed to improve the RDT sensitivity (RDT version N in Additional file 2). We therefore decided to use E1 as the target of the IC RDT. Comparison of sensitivities between versions B and E showed that gold-conjugated antibodies in version E yielded more aggregates at 10^7 pfu/mL of CHIKV, especially against Asian-genotype viruses. We therefore decided to stop further development of RDT version E.

**Fig. 2:**
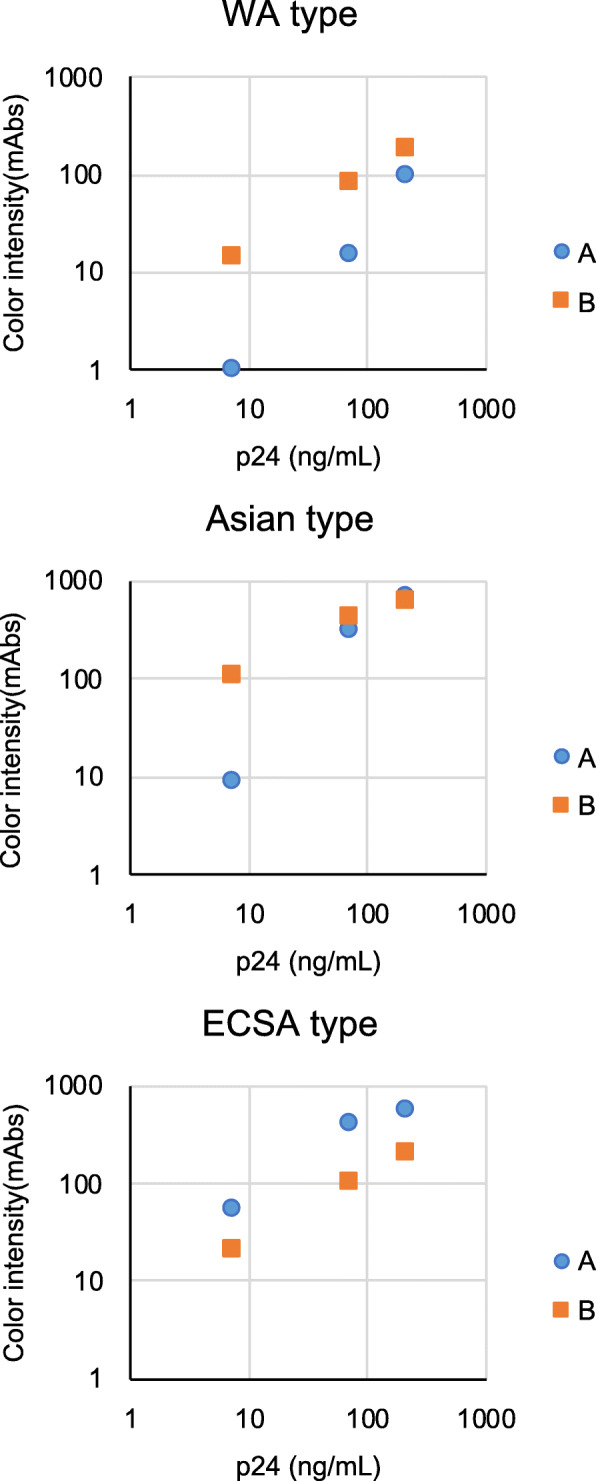
RDT reaction to pseudotyped lentiviruses bearing CHIKV envelope proteins. Pseudotyped lentiviruses bearing CHIKV envelope proteins from ECSA-genotype strain CP10, Asian-genotype strain CK12–686 and WA-genotype strain 37,997 were prepared at three concentrations of HIV-1 capsid protein (210, 70, and 7 ng/mL). Blue circles and orange squares indicate version-A and -B RDT measurements, respectively. The y-axis indicates the intensity of the test line (milli-absorbance units; mAbs)

We next examined the sensitivity of the version-B RDT to WA-genotype CHIKV. Since we do not have access to live CHIKV of the WA genotype, we used CHIKV envelope-pseudotyped lentivirus to evaluate the IC RDT sensitivity. As shown in Fig. 2, the version-B RDT showed higher sensitivity than did the 1st version RDT (version A) for detection of WA-genotype virus along with Asian-genotype virus. However, as we anticipated from the results shown in Fig. 1, sensitivity of the version-B RDT was not comparable to that of the version-A RDT for detection of ECSA-genotype CHIKV.

### Trial of 2nd-generation RDT version B with sera from Asian-genotype CHIKV patients

An Asian-genotype CHIKV outbreak occurred on the Caribbean island of Aruba in 2014–2015. We previously evaluated the version-A RDT using Aruban clinical samples of Asian-genotype CHIKV and found low sensitivity (33.3%) for these specimens [27]. We performed an evaluation of the sensitivity of our the new generation of the RDT with 80 samples (40 anti-CHIKV IgM- and IgG-negative, 40 anti-CHIKV IgM- or IgG-positive). Individual data for the Aruba island patients are summarized in Additional file 3. Among the 80 samples, only 5 CHIKV RT-PCR-positive and IgG- and IgM-negative samples had been obtained. The 2nd-generation RDT version B detected 4 out of 5 of these samples (80, 95% confidence interval (95%-CI) 37.6–96.4), as shown in Table 2. The sensitivity was lower in the IgG−/IgM-positive group (7 of 21 (33.3, 95%-CI 17.2–54.6)). Similarly, specificity was high (33 out of 35 (94.3, 95%-CI 81.4–98.4)) in the IgG- and IgM-negative group and lower in the IgG−/IgM-positive group (12 of 19 (63.2, 95%-CI 41.0–80.9)). The sensitivity and specificity did not change significantly in the 34 IgM-positive and 31 IgG-positive groups (Additional file 4).

**Table 2:** Evaluation of 2nd-generation CHIKV E1 detection RDT version B in clinical samples

IgG/IgM	PCR	IC RDT		Sensitivity	Specificity	OAA
		(+)	(−)			
(−)	(+)	4	1	80.0		92.5
	(−)	2	33		94.3	
(+)	(+)	7	14	33.3		47.5
	(−)	7	12		63.2	
Total	(+)	11	15	42.6		70.0
	(−)	9	45		83.3	

IgG/IgM (−): anti-CHIKV IgM- and IgG-negative cases

IgG/IgM (+): anti-CHIKV IgM- or IgG-positive cases

Sensitivity: percentage of matches between results of PCR and IC RDT in PCR positive samples

Specificity: percentage of matches between results of PCR and IC RDT in PCR negative samples

*OAA* overall agreement (percentage of total matches between results of PCR and IC RDT)

Of those 80 samples, a subset of 20 also was tested using the previous RDT version A in order to permit side-by-side comparison with the version-B RDT. These 20 (10 PCR-positive and 10 PCR-negative) samples comprised 6 that were IgG- and IgM-negative and 14 that were IgG- or IgM-positive (Additional files 3 and 5). The version-A RDT failed to detect any of these samples, while the version-B RDT detected 4 out of 10 PCR-positive samples (40%). Sensitivity and specificity of the version-B RDT for these 20 samples were comparable to those for the remaining 60 samples (Additional file 6).

### Sensitivity of the 3rd-generation IC RDT version O for ECSA- and Asian-genotype CHIKV isolates and CHIKV envelope-pseudotyped viruses

**Fig. 3:**
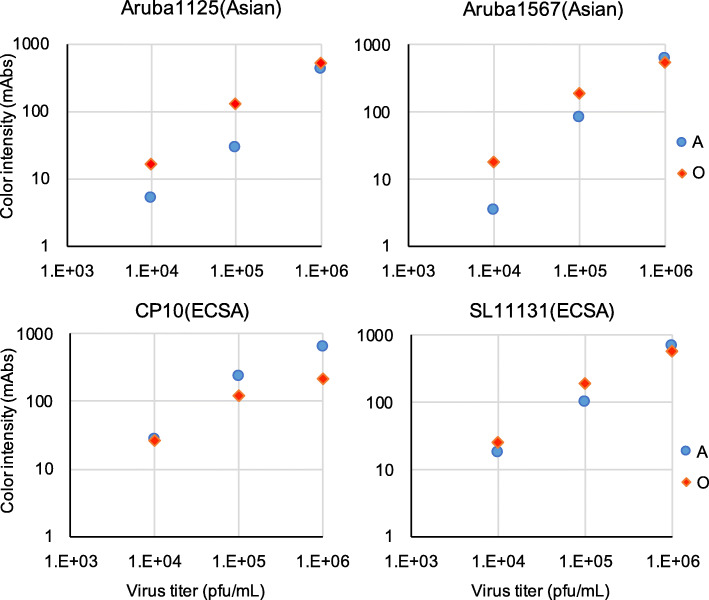
RDT reaction to CHIKV ECSA-genotype strains CP10 and SL11131 and Asian-genotype strains ARUBA-15801125 and ARUBA-15800567. The x-axis denotes viral titers in plaque forming units (PFU) per mL. Blue circles and red diamonds indicate version-A and -O RDT measurements, respectively. The y-axis indicates the intensity of the test line (milli-absorbance units; mAbs)

**Fig. 4:**
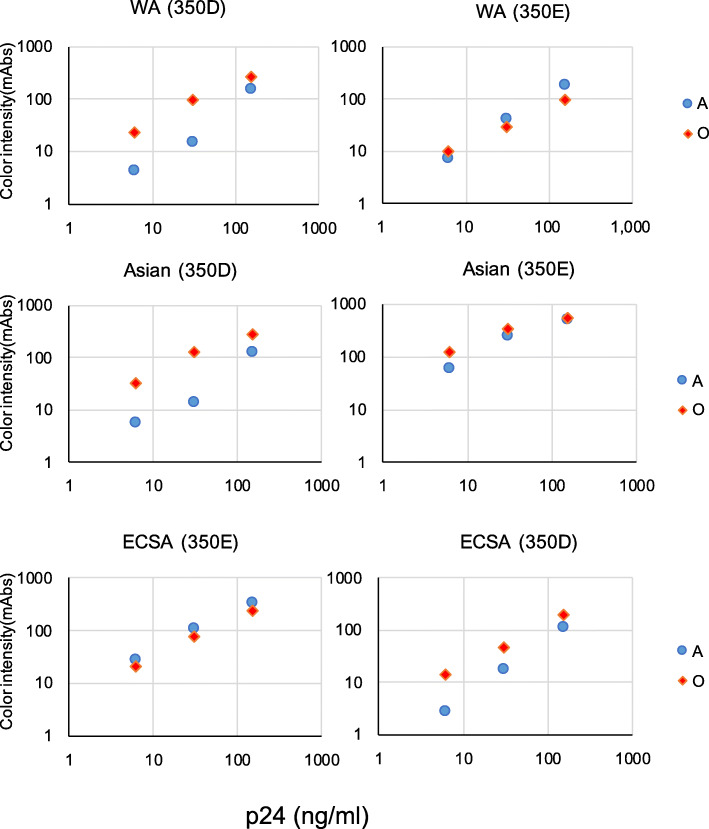
RDT reaction to pseudotyped lentiviruses bearing CHIKV envelope proteins with substitutions at residue 350. Pseudotyped lentiviruses bearing CHIKV envelope proteins from ECSA-genotype strain CP10, Asian-genotype strain CK12–686, WA-genotype strain 37,997, or mutant envelope proteins encoding glutamic acid-to-aspartic acid (CP10) or aspartic acid-to-glutamic acid (CK12–686 and 37,997) substitutions at residue 350 of the 6 K-E1 polyprotein were prepared at three concentrations of HIV-1 capsid protein (150, 30, and 6 ng/mL). Blue circles and red diamonds indicate version-A and -O RDT measurements, respectively. The y-axis indicates the intensity of the test line (milli-absorbance units; mAbs)

Although the version-B RDT showed improved sensitivity for Asian-genotype CHIKV, the sensitivity of the version-B RDT to ECSA-genotype CHIKV was not comparable to that of the version-A RDT (Figs. 1 and 2). To address this issue, we combined the antibodies used in the version-A and -B RDTs to generate a 3rd generation (designated version O) of the E1-detecting IC RDT. The detection limit of the version-O RDT for ECSA-genotype virus was comparable to that of the version-A RDT, both against live virus (1.0 × 10^4 pfu/mL) and against pseudotyped vectors (6 ng/mL of HIV-1 capsid protein) (Figs. 3 and 4). The improved sensitivity of the version-B RDT for Asian- and WA-genotype viruses was retained in the version-O RDT (Figs. 3 and 4).

As described previously, Asian-genotype CHIKV carries an aspartic acid (D) at position 350 of the 6 K-E1 protein, whereas certain ECSA-genotype CHIKVs carry a glutamic acid (E) at the corresponding position; this substitution is responsible for the decreased sensitivity of RDT version A for Asian-genotype CHIKVs [28]. The sensitivities of the 3rd generation of the E1 detection RDT (version O) to each genotype were not affected by this amino acid substitution (Fig. 4).

Furthermore, we did not detect any cross-reactivity of the version-O RDT with SINV at a titer 10^7/mL (7.8 mAbs), while the version-A RDT showed slight cross- reactivity at the same titer of SINV (43.9 mAbs).

### Trial of 3rd-generation RDT version O with CHIKV patient sera in Bangladesh

Since the volumes of sera from Aruba island, especially those of CHIKV PCR-positive ones, were limited, we decided to perform testing using clinical samples from other CHIKV-endemic areas. We accessed serum samples in Apollo Hospitals Dhaka, Bangladesh, where molecular detection of CHIKV and DENV was performed by RT-PCR screening during the 2017 CHIKV epidemic in Dhaka. These DENV positive sample sets were used for evaluating the specificity of the CHIKV detection RDT. As shown in Table 3, the version-O RDT exhibited 92% (92 out of 100) sensitivity and 100% (100 out of 100) specificity in this setting. Among 210 CHIKV RT-PCR-negative and DENV RT-PCR-negative samples identified in Dhaka, 104 were anti-CHIKV IgM positive. We further investigated 93 of these 104 anti-CHIKV IgM-positive cases and found that three (3%) yielded positive results using our E1-antigen IC RDT (Additional file 7).

**Table 3:** Evaluation of 3rd-generation CHIKV E1 detection RDT version O in clinical samples

PCR	IC RDT		Sensitivity	Specificity	OAA
	(+)	(−)			
(+)^a^	92	8	92		96
(−)^b^	0	100		100	

Sensitivity: percentage of matches between results of PCR and IC RDT in PCR positive samples

Specificity: percentage of matches between results of PCR and IC RDT in PCR negative samples

*OAA* overall agreement

^a^ 99 anti-CHIKV IgM-negative samples and one anti-CHIKV IgM-positive sample

^b^dengue PCR-positive samples

## Discussion

In the present study, using newly obtained MAbs [29], we developed new IC CHIKV-E1 antigen detection RDTs with improved sensitivity to Asian- and WA-genotype viruses compared to the previous version-A RDT [28]. Although we tested only 5 samples of Asian-genotype CHIKV-infected cases (i.e., Aruban cases before anti-CHIKV IgM/IgG seroconversion), the 2nd generation CHIKV antigen detection RDT version B yielded positive results in 4 of these 5 samples (i.e., 80% sensitivity). Sample numbers are low in this Asian-CHIKV serum panel, but the results suggest increased sensitivity over the previous version of RDT [27]. Furthermore, the 3rd generation RDT version O showed sensitivity of 92% (92 out of 100, 95%-CI 85.0–95.9) in sera from 100 ECSA-genotype CHIKV-infected patients from Bangladesh. The version-O RDT also showed higher sensitivity to cultured Asian- and WA genotype CHIKVs than the version-A RDT did. These results suggest that the version-O RDT will be better for worldwide application compared to the version-A and -B RDTs.

As described in Materials and Methods, the sampling dates after the onset of fever were median 7 days and the median Ct value and its IQR of CHIKV-detecting PCR were 35.29 (34.22–35.50) for the Aruba samples. The sampling days after the onset of fever did not differ significantly among 11 PCR-positive and RDT-positive (median 6 days), 15 PCR-positive and RDT-negative (median 7 days), and 9 PCR-negative and RDT-positive groups (median 7 days). Importantly, many of the samples from Aruba were collected after anti-CHIKV antibody seroconversion had occurred. We look forward to confirming our findings in a larger sample size of Asian-genotype CHIKV-infected patients, preferably before seroconversion. After seroconversion, detection of anti-CHIKV antibodies can be used for diagnosis. Therefore, it would be ideal to simultaneously detect both CHIKV antigen and anti-CHIKV antibodies for accurate diagnosis.

In terms of the false-positive rate in CHIKV PCR-negative samples, the specificity of the version-O RDT in samples in Bangladesh was 100%, while that of the version-B RDT was 83.3% (45 out of 54) in samples from Aruba. Specificity of the version-B RDT in the Aruba samples was 94.2% (33 out of 35) for anti-CHIKV IgM- and IgG-negative cases, while that in anti-CHIKV IgM- or IgG-positive cases was 63.2% (12 out of 19) (Table 2). As described above, the version-B RDT uses 3 clones of anti-CHIKV MAbs, while the version-O RDT uses 5 MAbs, including 3 MAbs used in the version-B RDT. Thus, it is unlikely that a specific component present only in the version-B RDT caused the false-positive reactions. Most of the Ct-values of CHIKV-detecting RT-PCR in Bangladesh samples were lower than 20, and the median Ct-value and its IQR was 17.02 (14.89–19.92) (see Additional file 7). Of course, it is not appropriate to directly compare Ct-values between Aruba and Dhaka patients given that these values were determined with different real-time PCR systems, but these Ct-values suggest that the CHIKV sera in Dhaka were collected earlier in the disease course. Results of ELISA detecting anti-CHIKV IgM also support this idea, since only one out of 100 CHIKV PCR-positive cases was anti-CHIKV IgM positive (Table 3, Additional file 7). In contrast, among 54 PCR-negative samples from Aruba, 19 were positive for either anti-CHIKV IgM or IgG (Table 2, Additional file 3). As the kinetics of chikungunya antigenemia are not well understood, it is possible that the IC RDT-positive but PCR-negative samples are not truly CHIKV negative. In other words, CHIKV structure proteins might have persisted longer than the viral genome in blood, since the structural proteins might be expressed from a sub-genomic or defective viral RNA without full-length genomic RNA replication. In fact, persistence of a defective alphavirus genome in infected cells has been reported previously [37]. Indeed, three (3%) positive results were obtained when we tested 93 CHIKV PCR-negative and DENV PCR-negative but anti-CHIKV IgM-positive samples (Additional file 7).

The precise mechanisms associated with chronic CHIKV-associated joint disease are largely unknown, although the effects of abnormalities present prior to infection cannot be ruled out. In CHIKV-infected patients, high viral loads are common, and anti-CHIKV IgM or IgG has been observed to persist in patients for 18 months or longer [38, 39]. These findings suggest that continuous immune stimulation, possibly by persistent or continuously expressed CHIKV antigens, could play a role in prolonged CHIKV-associated poly-arthralgia, although no tools are available to rapidly detect CHIKV antigens. It will be interesting to use the diagnostic RDT described in the present work for follow-up studies on antigen persistence in post-chikungunya chronic poly-arthralgia. Thus, it will be critical to further evaluate these false-positive cases using our revised CHIKV RDT.

## Conclusion

We developed a new CHIKV E1-antigen rapid diagnostic test with improved sensitivity (compared to the previous version) to the Asian and WA genotypes of CHIKV. Although the revised RDT needs to be validated against larger panels of sera with varying CHIKV genotypes, the new RDT holds a promise for use in endemic areas for ECSA-genotype CHIKV encoding a 6 K-E1 protein with an E350 residue, viruses that are currently spreading from South Asia to South East Asia [36, 40].

## Supplementary information

**Additional file 1.** Selection of monoclonal antibodies (MAbs) for CHIKV detection rapid diagnostic test. (A) Means and standard deviations of signal/background ratios for selection of MAbs against E1 protein. AuNPs column indicates MAb conjugated with AuNPs and membrane column indicates MAb adsorbed onto nitrocellulose membrane. Green: Signal/background ratio was lower than 1.0 (strong non-specific reaction or no signal). Orange: Signal/background ratio was between 1.0 and 2.0 (faint). Red: Signal/background ratio was higher than 2.0 (strong positive). Yellow: Signal/background ratio was higher than 2.0 but standard deviation was high. (B) Means of signal/background ratios for selection of MAbs against capsid protein. AuNPs column indicates MAb conjugated with AuNPs and membrane column indicates MAb adsorbed onto nitrocellulose membrane. Green: Signal/background ratio was lower than 1.0 (strong non-specific reaction or no signal). Orange: Signal/background ratio was between 1.0 and 2.0 (faint). Red: Signal/background ratio was higher than 2.0 (strong positive)

**Additional file 2.** RDT reaction to CHIKV Asian-genotype and ECSA-genotype strains. ECSA-genotype strain CP10 and Asian-genotype strains ARUBA-15801567 (ARUBA1567) and ARUBA-15801125 (ARUBA1125) were grown in Vero cells. The x-axis denotes viral titer in plaque forming units (PFU) /mL. Blue circles, orange squares, and gray diamonds indicate CP10, ARUBA1125, and ARUBA1567 measurements, respectively. The y-axis indicates the intensity of the test line (milli-absorbance units; mAbs).

**Additional file 3.** Data of Aruba patients. ND: not determined.

**Additional file 4.** Evaluation of CHIKV E1 detection RDT version B in anti-CHIKV IgM or IgG-positive clinical samples. CHIKV E1 detection RDT version B were evaluated in 34 anti-CHIKV IgM-positive and 31 IgG-positive clinical samples. OAA: overall agreement.

**Additional file 5.** Comparison of CHIKV E1 detection RDT versions A and B in 20 clinical samples. OAA: overall agreement.

**Additional file 6.** Evaluation of CHIKV E1 detection RDT version B in 60 clinical samples. OAA: overall agreement.

**Additional file 7.** Data of Dhaka patients. ND: not determined. E1(mAbs): mili absorbance units of CHIKV E1 antigen immunochromatogaraphic rapid diagnostic test (version O). Positive results in CHIKV E1 detection, anti-CHIKV IgM, dengue virus NS1, anti-dengue virus IgM and IgG are highlighted with red.

## Data Availability

All data generated or analyzed during this study are included in this published article and its supplementary information files.

## Abbreviations

CHIKV: Chikungunya virus
ECSA: East/Central/ South African
WA: West African
IC: Immunochromatorgraphic
RDT: Rapid diagnostic test
MAb: Monoclonal antibody
SINV: Sindbis virus
DENV: Dengue virus
IOL: Indian Ocean lineage
mAbs: Milli-absorbance units
RT-PCR: Reverse transcription-polymerase chain reaction
OAA: Overall agreement
