# Supplementary material for: Promising application of monoclonal antibody against chikungunya virus E1-antigen across genotypes in immunochromatographic rapid diagnostic tests
Source: Virol J. 2020 Jul 2;17:90. doi: 10.1186/s12985-020-01364-4 (PMC7330967; doi:10.1186/s12985-020-01364-4)
Supplement: Supplementary file 1 — Additional file 1. Selection of monoclonal antibodies (MAbs) for CHIKV detection rapid diagnostic test. (A) Means and standard deviations of signal/background ratios for selection of MAbs against E1 protein. AuNPs column indicates MAb conjugated with AuNPs and membrane column indicates MAb adsorbed onto nitrocellulose membrane. Green: Signal/background ratio was lower than 1.0 (strong non-specific reaction or no signal). Orange: Signal/background ratio was between 1.0 and 2.0 (faint). Red: Signal/background ratio was higher than 2.0 (strong positive). Yellow: Signal/background ratio was higher than 2.0 but standard deviation was high. (B) Means of signal/background ratios for selection of MAbs against capsid protein. AuNPs column indicates MAb conjugated with AuNPs and membrane column indicates MAb adsorbed onto nitrocellulose membrane. Green: Signal/background ratio was lower than 1.0 (strong non-specific reaction or no signal). Orange: Signal/background ratio was between 1.0 and 2.0 (faint). Red: Signal/background ratio was higher than 2.0 (strong positive) [file 12985_2020_1364_MOESM1_ESM.pdf]

A

		Membrane						
	Ave	3D11	9E3	11F11	13H11	19B8	RC-5	15B2
AuNps	3D11		1.1 ±0.1	1.0 ±0.3	0.7 ±0.4	0.6 ±0.5	0.8 ±0.5	1.0 ±0.3
	9E3	1.5 ±0.3		1.3 ±0.2	1.0 ±0.5	0.6 ±0.6	0.8 ±0.3	1.4 ±0.3
	11F11	1.1 ±0.4	1.1 ±0.4		1.4 ±0.3	0.7 ±0.5	1.1 ±0.0	1.9 ±0.6
	13H11	2.1 ±0.5	1.2 ±0.1	0.9 ±0.2		0.6 ±0.3	0.8 ±0.4	2.1 ±0.9
	19B8	1.3 ±0.4	0.7 ±0.5	0.8 ±0.5	0.5 ±0.4		0.5 ±0.3	1.0 ±0.2
	RC-5	1.1 ±0.7	1.0 ±0.4	1.2 ±0.2	1.0 ±0.4	0.9 ±0.5		3.6 ±3.8

Mean ±SD

(green)	Strong non specific reaction or No signal	< 1.0
(orange)	Faint	1.0 - 2.0
(red)	Strong positive	>2.0
(yellow)	Strong positive but high standard deviation	

B

		Membrane				
		24B3	26A2	32A3	37C7	41G5
AuNps	24B3		5.4	2.4	1.8	12.3
	26A2	12.0		15.5	11.9	9.2
	32A3	1.0	8.4		1.5	10.2
	37C7	1.2	4.3	2.4		6.2
	41G5	46.8	4.2	28.5	5.9	

(green)	Strong non specific reaction	< 1.0
(orange)	Faint	1.0 - 2.0
(red)	Strong positive	>2.0
