# Supplementary figures and images for: Promising application of monoclonal antibody against chikungunya virus E1-antigen across genotypes in immunochromatographic rapid diagnostic tests
Source: Virol J. 2020 Jul 2;17:90. doi: 10.1186/s12985-020-01364-4 (PMC7330967; doi:10.1186/s12985-020-01364-4)

Version-E RDT

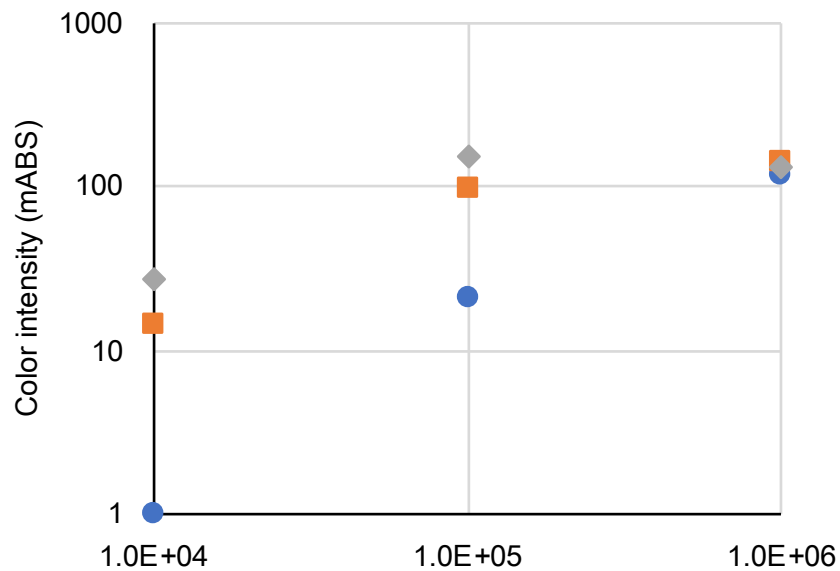

Version-F RDT

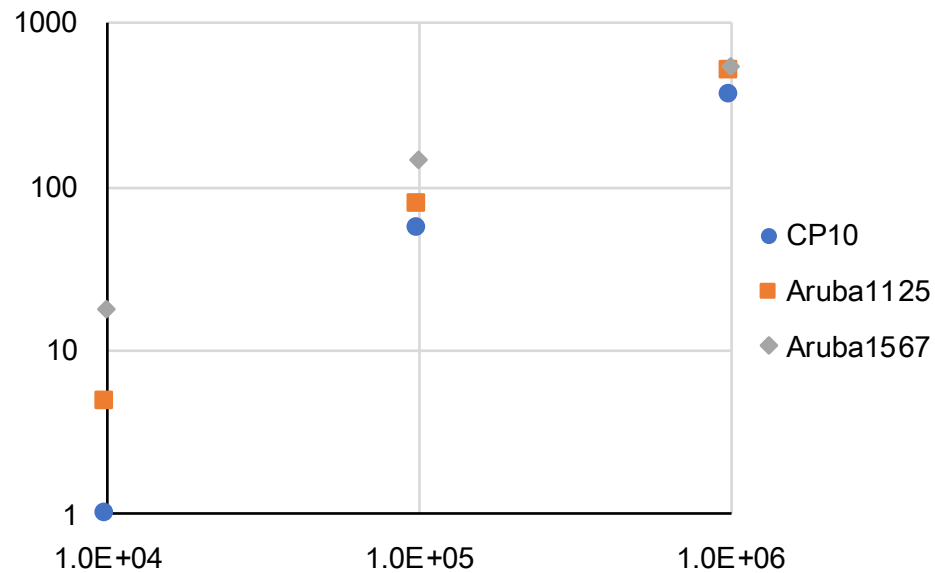

Version-M RDT

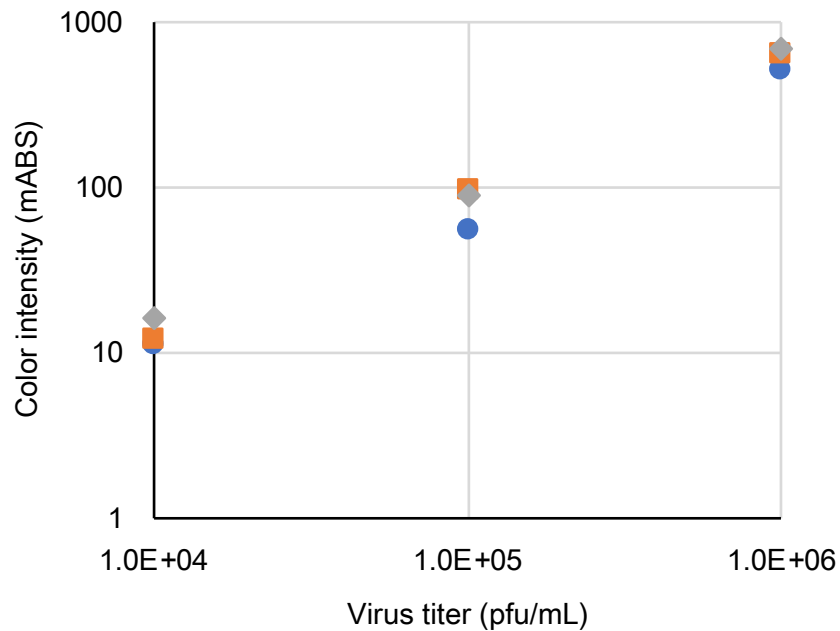

Version-N RDT

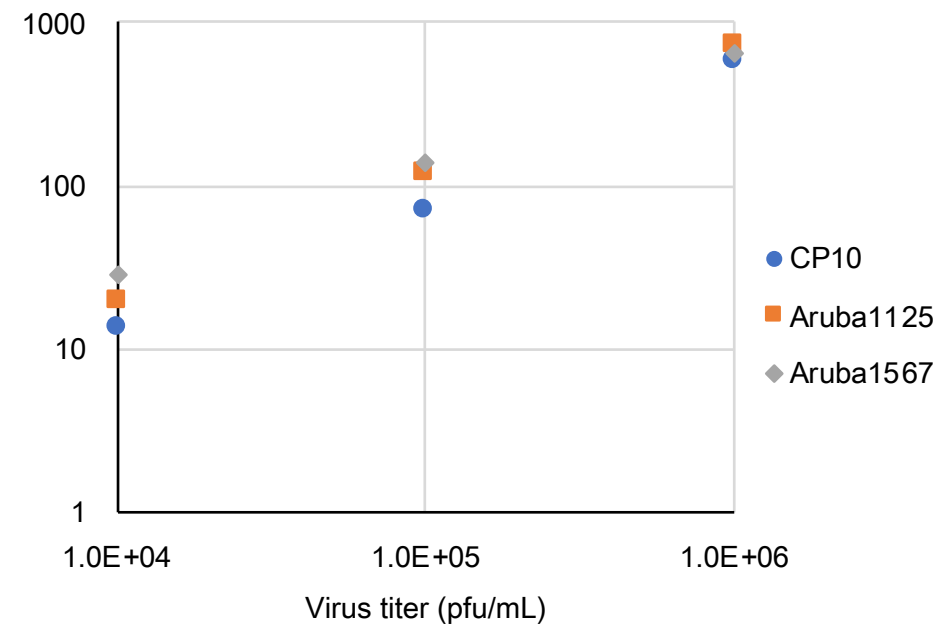

Supplement: Supplementary file 2 — Additional file 2. RDT reaction to CHIKV Asian-genotype and ECSA-genotype strains. ECSA-genotype strain CP10 and Asian-genotype strains ARUBA-15801567 (ARUBA1567) and ARUBA-15801125 (ARUBA1125) were grown in Vero cells. The x-axis denotes viral titer in plaque forming units (PFU) /mL. Blue circles, orange squares, and gray diamonds indicate CP10, ARUBA1125, and ARUBA1567 measurements, respectively. The y-axis indicates the intensity of the test line (milli-absorbance units; mAbs). [file 12985_2020_1364_MOESM2_ESM.pdf]
